# Papillary Thyroid Carcinoma in a Pediatric Patient With Graves’ Disease: A Case Report and Literature Review

**DOI:** 10.7759/cureus.104829

**Published:** 2026-03-07

**Authors:** Elisavet Kanna, Despina Panayiotou, Alexandros Ganaiem, Elpis Vlachopapadopoulou, Ioannis Skondras

**Affiliations:** 1 Second Pediatric Surgery Department, Panagiotis and Aglaia Kyriakou Children's Hospital, Athens, GRC; 2 Otolaryngology Department, Panagiotis and Aglaia Kyriakou Children's Hospital, Athens, GRC; 3 Otolaryngology - Head and Neck Surgery Department, Sotiria Thoracic Diseases Hospital of Athens, Athens, GRC; 4 Endocrinology Department, Panagiotis and Aglaia Kyriakou Children's Hospital, Athens, GRC

**Keywords:** autoimmune hyperthyroidism, graves’ disease, papillary thyroid carcinoma, thyroidectomy, thyroid nodule, thyroid nodule management

## Abstract

Graves’ disease (GD) is the most common cause of hyperthyroidism in children and adolescents; however, its coexistence with differentiated thyroid carcinoma is rare and may pose significant diagnostic challenges, as diffuse hyperplasia and hypervascularity can obscure malignant nodules. We report the case of a 15-year-old girl with GD treated with methimazole who was found on follow-up ultrasonography to have a hypoechoic thyroid nodule with punctate calcifications and peripheral vascularity. Although she was biochemically euthyroid, fine-needle aspiration cytology was consistent with papillary thyroid carcinoma (Bethesda V), and cervical lymph node mapping showed no evidence of metastatic disease. The patient underwent total thyroidectomy without complications and was discharged on the second postoperative day without the need for calcium supplementation. Histopathological examination confirmed an 11-mm papillary thyroid carcinoma, classic variant, arising in a background of GD, without capsular or vascular invasion. At nine months of follow-up, she remained clinically stable and biochemically euthyroid on levothyroxine, with no evidence of recurrence. This case highlights the importance of careful ultrasonographic surveillance and timely cytological evaluation of thyroid nodules in pediatric patients with GD and supports total thyroidectomy as a safe and definitive treatment when malignancy is suspected.

## Introduction

Graves’ disease (GD) is the most common cause of hyperthyroidism in children and adolescents, resulting from autoimmune stimulation of the thyroid-stimulating hormone receptor (TSHR) by stimulating antibodies (TSHR antibodies, TRAb) [1]. Although the disorder is typically characterized by diffuse thyroid hyperplasia and increased vascularity, differentiated thyroid carcinoma (DTC) may occasionally coexist, creating diagnostic and therapeutic challenges [2].

In adults, several studies have demonstrated a higher prevalence of thyroid nodules and malignancy among patients with GD, with reported rates ranging from 0.5% to 15% [3]. Most of these malignancies are papillary thyroid carcinomas (PTCs), and while many are discovered incidentally after thyroidectomy, some exhibit more aggressive features such as lymph node metastases and extrathyroidal extension. The pathophysiological mechanisms linking GD to thyroid carcinogenesis remain uncertain but may involve chronic TSH receptor stimulation, autoimmune inflammation, or growth-promoting effects of thyroid-stimulating antibodies.

In contrast, in the pediatric population, the coexistence of GD and thyroid carcinoma is extremely rare, yet when it occurs, it may follow a more aggressive clinical course than in adults. Pediatric data are limited to a few retrospective studies and isolated case reports, which suggest that careful evaluation of thyroid nodules in children with GD is crucial, as malignancy can be easily overlooked due to the hypervascular and diffusely enlarged nature of the gland [4].

The objective of this report is to present a rare pediatric case of concurrent GD and PTC, and to discuss possible pathophysiological mechanisms, diagnostic challenges, and management strategies.

## Case presentation

**Figure 1:**
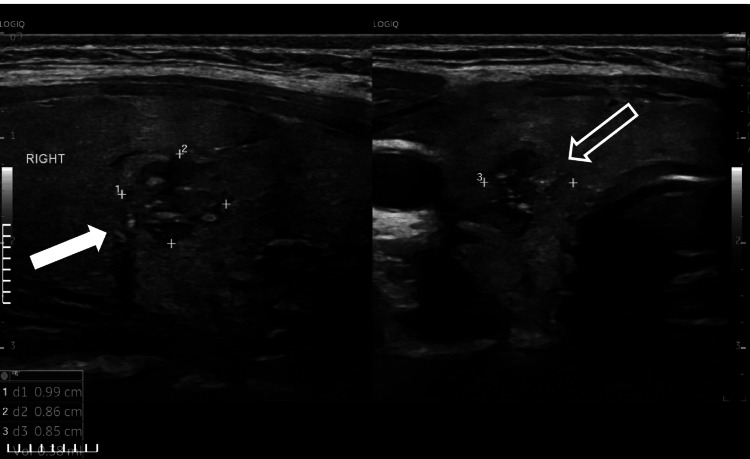
Thyroid ultrasonography demonstrating a hypoechoic solid nodule measuring approximately 1 cm in the right thyroid lobe. The solid arrow indicates the hypoechoic thyroid nodule, while the open arrow highlights punctate echogenic foci (microcalcifications), which are sonographic features suspicious for papillary thyroid carcinoma.

A 15-year-old adolescent girl was referred to the Endocrinology Department for investigation of a thyroid nodule. The girl had been diagnosed with GD one year earlier and had been treated with methimazole (10 mg/day orally) for approximately 15 months under the care of a private endocrinologist, with good compliance. At follow-up, a thyroid sonogram revealed a hypoechoic nodule in the middle of the right lobe, measuring approximately 1 cm with punctate calcifications and peripheral vascularity (Figure 1).

On clinical examination, the thyroid gland was palpable, with mildly enlarged dimensions but without a discrete nodule. Her pulse rate and blood pressure were normal, and there was no tremor or other signs of thyrotoxicosis. Laboratory tests demonstrated a euthyroid state, with the following results: thyroid-stimulating hormone (TSH) 0.721 μIU/mL (reference range 0.4-4.0), free thyroxine (FT4) 1.55 ng/dL (reference range 0.8-1.8), and free triiodothyronine (FT3) 3.12 pg/mL (reference range 2.3-4.2).

Given the sonographic features suggestive of possible malignancy and the known increased risk of carcinoma in patients with GD, a fine-needle aspiration (FNA) of the nodule was performed. Cytological evaluation was consistent with PTC (Bethesda category V). A comprehensive cervical lymph node ultrasound mapping showed no evidence of lymph node involvement. Based on these findings, a total thyroidectomy was recommended.

**Figure 2:**
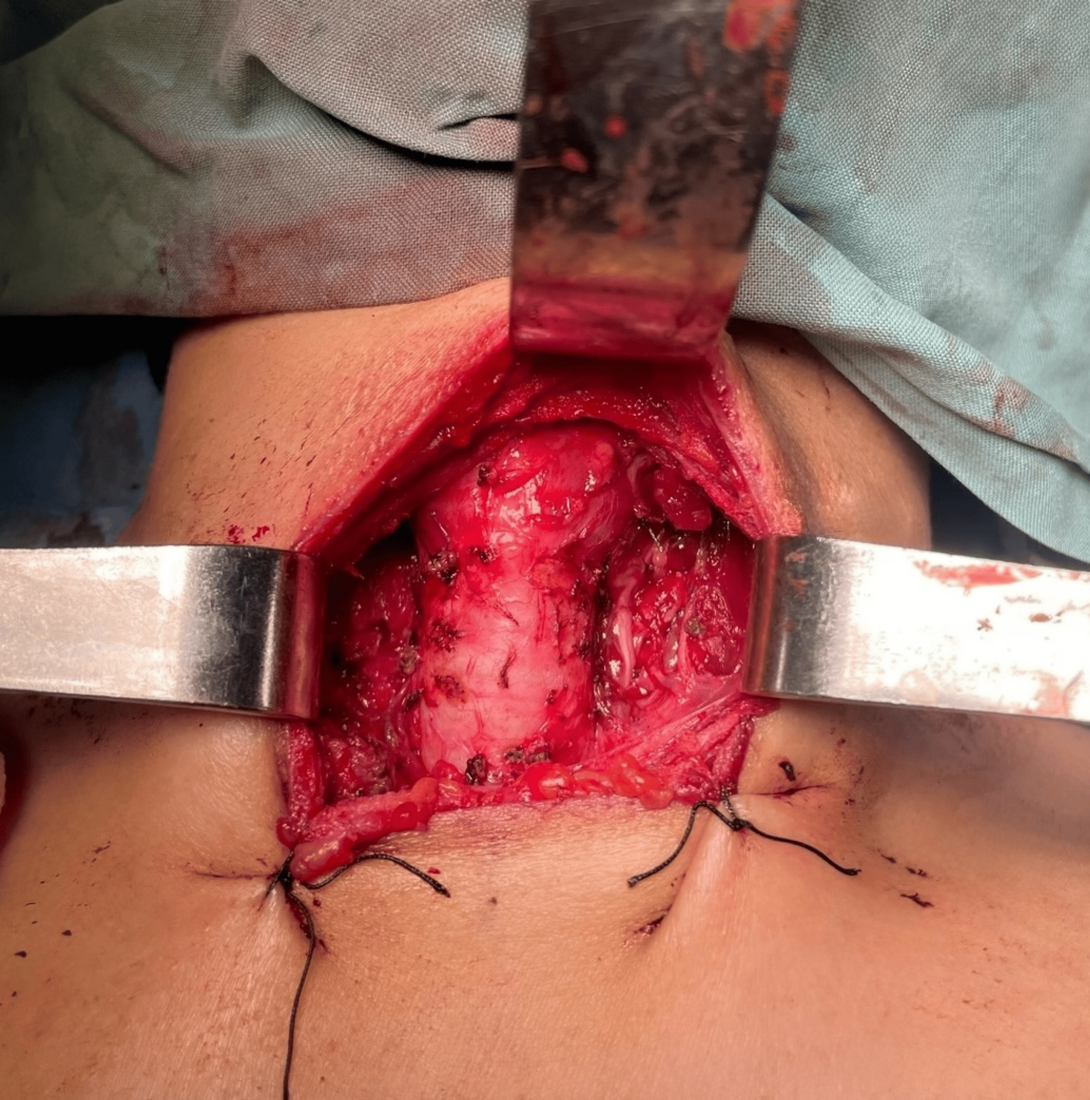
Intraoperative view of the surgical field immediately after total thyroidectomy, demonstrating a clean operative bed with adequate hemostasis and preservation of the parathyroid glands and recurrent laryngeal nerves.

A total thyroidectomy with preservation of the parathyroid glands was performed without complications. There was no intraoperative hemorrhage and no injury to the recurrent laryngeal nerves (Figure 2). Hemostasis was secure, and the postoperative recovery was smooth. The patient was discharged on the second postoperative day without requiring oral calcium supplementation.

Microscopic examination of the resected thyroid gland revealed features consistent with PTC, classic variant, arising in a background of diffuse hyperplasia typical of GD. The tumor displayed papillary and follicular architectural patterns, lined by epithelial cells with nuclear enlargement, overlapping, chromatin clearing (“Orphan Annie eye” nuclei), nuclear grooves, and occasional intranuclear pseudoinclusions. Psammoma bodies were also observed within the fibrovascular cores.

The carcinoma measured 11 mm in greatest dimension, was well-circumscribed, and showed no evidence of capsular or vascular invasion. The adjacent thyroid tissue demonstrated diffuse follicular hyperplasia, lymphocytic infiltration, and colloid scalloping, consistent with GD. No lymph node metastases were identified.

At nine months of follow-up, the patient remained clinically stable and biochemically euthyroid on levothyroxine replacement therapy, with no evidence of recurrence on cervical ultrasound. However, a longer follow-up is required to fully assess long-term oncologic outcomes.

**Figure 3:**
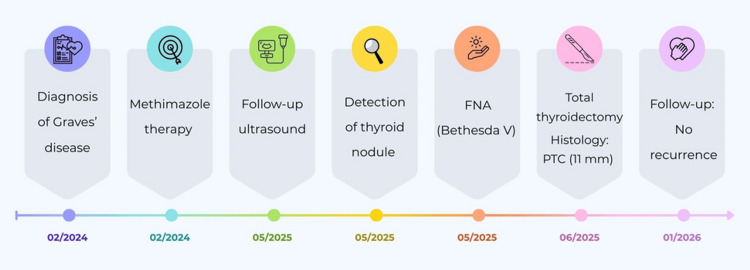
Timeline of the clinical course and management from the diagnosis of Graves’ disease to total thyroidectomy, histological confirmation of papillary thyroid carcinoma (PTC), and postoperative follow-up. FNA: fine-needle aspiration Source: The figure was created by the authors using Canva design software (Canva Pty Ltd., Sydney, Australia).

The clinical course and key management steps are summarized in Figure 3.

## Discussion

GD is the most common cause of hyperthyroidism in children and adolescents. Pediatric cases, however, represent only approximately 1-5% of all patients with GD. An important clinical consideration in this setting is the potential presence of thyroid nodules, which may harbor malignancy. Ultrasonographic studies have reported thyroid nodules in 40-60% of pediatric patients with GD [4]. The marked hypervascularity and diffuse enlargement characteristic of GD may further hinder the detection of such nodules, contributing to delayed diagnosis.

In this context, careful ultrasonographic evaluation is essential. High-risk ultrasound features suggestive of malignancy in pediatric thyroid nodules, as defined by international guidelines, are summarized in Table 1.

**Table 1:** Ultrasound features suggestive of malignancy in pediatric thyroid nodules according to international guidelines. Based on risk stratification criteria from the American Thyroid Association (ATA) and European Thyroid Association (ETA) guidelines for pediatric thyroid nodules. FNA: fine-needle aspiration

No.	Ultrasound feature
1	Hypoechogenicity
2	Solid or predominantly solid composition
3	Punctate microcalcifications
4	Irregular or infiltrative margins
5	Taller-than-wide shape
6	Increased intranodular or peripheral vascularity
7	Abnormal cervical lymph nodes
8	Nodule ≥1 cm with suspicious features (indication for FNA)

Among children with GD who undergo thyroidectomy, approximately 22% have been found to have DTC, most commonly PTC [4]. This prevalence is notably higher than in the general pediatric population and underscores the need for thorough preoperative evaluation, including high-resolution ultrasound and FNA of suspicious lesions [5]. Individual case reports further support this association: Tuli et al. described papillary microcarcinoma in a 16-year-old boy with GD, while Kojima-Ishii et al. reported minimally invasive follicular carcinoma in a 14-year-old with the same underlying condition [2,3]. Collectively, these findings suggest that the coexistence of GD and thyroid carcinoma in children is not incidental but may reflect shared biological mechanisms. Additionally, Kovatch et al. examined the prevalence of DTC in pediatric GD and highlighted the importance of preoperative ultrasound in selecting appropriate definitive therapy [4].

Previously reported pediatric cases of GD associated with thyroid carcinoma are summarized in Table 2.

**Table 2:** Summary of reported pediatric cases of Graves’ disease associated with thyroid carcinoma. PTC: papillary thyroid carcinoma; RAI: radioactive iodine

Author	Age/Sex	Carcinoma Type	Tumor Size	Treatment	Outcome	Follow-Up
Kojima-Ishii et al. [3]	14/F	Follicular carcinoma	20 mm	Total thyroidectomy	No recurrence	12 months
Tuli et al. [2]	16/M	Papillary carcinoma	6 mm	Total thyroidectomy	No recurrence	6 months
Kovatch et al. [4]	Pediatric series	PTC predominant	Variable	Surgery/RAI	No recurrence	Variable
Present case	15/F	PTC	11 mm	Total thyroidectomy	No recurrence	9 months

Several mechanisms have been proposed to explain the coexistence of GD and DTC in children, including chronic stimulation of the TSHR by thyroid-stimulating antibodies and autoimmune inflammation. Although a definitive causal relationship has not been established, the hyperplastic environment of the Graves’ thyroid gland may favor tumor development.

The management of pediatric GD is guided by international recommendations. According to the European Thyroid Association (ETA), methimazole or carbimazole is recommended as first-line therapy, with beta-blockers used for symptomatic relief. Definitive therapy should be considered in cases of persistent hyperthyroidism, intolerance to antithyroid drugs, large goiters, or when thyroid nodules raise suspicion of malignancy. In such scenarios, total thyroidectomy is preferred over radioactive iodine in younger patients when definitive treatment is indicated [6]. In our patient, definitive surgical treatment was prompted by the presence of a suspicious thyroid nodule with cytology consistent with PTC.

Compared with adults, children with GD-associated DTC may more frequently present with microcarcinomas and occasionally with more aggressive histologic variants, such as the tall-cell variant. Pediatric patients may also present with regional lymph node involvement or lymphovascular invasion at diagnosis. Despite this, long-term prognosis in children is generally excellent, with high survival rates, which may reflect biological tumor behavior and the effectiveness of multidisciplinary pediatric management strategies.

Although thyroidectomy is a well-established treatment option for selected pediatric thyroid disorders, it carries a higher risk of postoperative complications in children compared with adults, particularly hypocalcemia secondary to hypoparathyroidism and recurrent laryngeal nerve injury. International guidelines emphasize that appropriate surgical expertise is essential to minimize perioperative complications in pediatric thyroid surgery, with meticulous dissection and preservation of the recurrent laryngeal nerves and parathyroid glands [7]. In the present case, total thyroidectomy proceeded without complications, and the patient’s postoperative recovery was smooth, consistent with evidence demonstrating that surgeon expertise is a key determinant of perioperative safety and functional outcomes.

Given the increased risk of malignancy in pediatric GD, routine ultrasound surveillance of the thyroid gland is recommended. Suspicious nodules should undergo preoperative FNA to guide management decisions. When malignancy is confirmed or strongly suspected, total thyroidectomy provides definitive treatment of both hyperthyroidism and thyroid carcinoma and facilitates postoperative surveillance with serum thyroglobulin levels. Postoperative follow-up should include periodic thyroid function testing, serum thyroglobulin measurement, and cervical ultrasonography to detect recurrence or persistent disease.

This case is noteworthy because it highlights the rare but clinically significant coexistence of GD and PTC in the pediatric population. It emphasizes the importance of systematic ultrasonographic surveillance even in biochemically controlled patients and supports guideline-based surgical management when malignancy is suspected.

Given the rarity of this association in children, continued reporting of similar cases may help improve understanding of the relationship between GD and thyroid carcinoma in the pediatric population.

## Conclusions

Although rare, the coexistence of GD and PTC can occur in the pediatric population and may present significant diagnostic challenges due to the hypervascular and diffusely enlarged thyroid gland. This case underscores the importance of systematic ultrasound evaluation and timely FNA of suspicious nodules in children with GD. Adherence to international guidelines and management by a multidisciplinary team are essential to ensure optimal outcomes. When malignancy is suspected or confirmed, total thyroidectomy provides definitive treatment for both conditions and is associated with excellent short-term and long-term outcomes.
